# Death of Dr. C. Robin

**Published:** 1886-08

**Authors:** 


### Death of Dr. C. Robin.

The Faculty of Medicine of Paris has just sustained a great loss in the death of Monsieur Charles Robin, Professor of Histology, which took place at Yosseron, in the Department of the Ain. He was suddenly seized with an attack of apoplexy, which carried him off in a few hours. He was sixty-four years of age, was a member of the Institute and of the Academy of Medicine, also a Senator for his Department.

Ex-King Theebaw's famous hairy family, which he long kept jealously at Mandalay, are coming to Europe for exhibition. The family have been renowned in Burmese history for many years, and the present members, a mother and son, form the fourth generation known. The mother is 63, quite blind, and usually sits motionless on a platform, occasionally fanning herself and speaking in a low, sweet voice. She was seen and described by Colonel Yule when on a mission to the court of Ava in 1855. Save her hands and feet, she is covered with long soft hair, like her son, who is covered even to the drums of his ears, the hair in some places being five inches long. The man is of medium height, with pale brown skin, and is fairly friendly, having been partly educated and married to a maid of honor. Neither he nor his mother have either canine teeth or grinders.

